# Transcriptional and Proteolytic Regulation of the Toxin-Antitoxin Locus *vapBC10* (*ssr2962/slr1767*) on the Chromosome of *Synechocystis* sp. PCC 6803

**DOI:** 10.1371/journal.pone.0080716

**Published:** 2013-11-19

**Authors:** Degang Ning, Shuibing Liu, Weidong Xu, Qiang Zhuang, Chongwei Wen, Xiaoxia Tang

**Affiliations:** 1 Department of Environment Sciences, School of the Environment, Jiangsu University, Zhenjiang, Jiangsu, China; 2 Department of Pharmaceutical engineering, School of Pharmacy, Jiangsu University, Xuefu Road, Zhenjiang, Jiangsu, China; University of Manchester, United Kingdom

## Abstract

VapBC toxin-antitoxin (TA) systems are defined by the association of a PIN-domain toxin with a DNA-binding antitoxin, and are thought to play important physiological roles in bacteria and archaea. Recently, the PIN-associated gene pair PIN-COG2442 was proposed to encode VapBC-family TA system and found to be abundant in cyanobacteria. However, the features of these predicted TA loci remain under investigation. We here report characterization of the PIN-COG2442 locus *vapBC10* (*ssr2962/slr1767*) on the chromosome of *Synechocystis* sp. PCC 6803. RT-PCR analysis revealed that the *vapBC10* genes were co-transcribed under normal growth conditions. Ectopic expression of the PIN-domain protein VapC10 caused growth arrest of *Escherichia coli* that does not possess *vapBC* TA locus. Coincidentally, this growth-inhibition effect could be neutralized by either simultaneous or subsequent production of the COG2442-domain protein VapB10 through formation of the TA complex VapBC10 *in vivo*. In contrast to the transcription repression activity of the well-studied antitoxins, VapB10 positively auto-regulated the transcription of its own operon via specific binding to the promoter region. Furthermore, *in vivo* experiments in *E. coli* demonstrated that the *Synechocystis* protease ClpXP2s, rather than Lons, could cleave VapB10 and proteolytically activate the VapC10 toxicity. Our results show that the PIN-COG2442 locus *vapBC10* encodes a functional VapBC TA system with an alternative mechanism for the transcriptional auto-regulation of its own operon.

## Introduction

Bacterial toxin-antitoxin (TA) systems are complexes of a stable toxic protein and its unstable inhibitor, which are typically encoded by a bicistronic operon. TA loci were originally found in low-copy-number plasmids and characterized as addiction modules to stabilize them by post-segregational killing [1]. Since then, such genetic modules are also discovered to be strikingly abundant and diverse on bacterial and archaeal chromosomes [2]–[4]. Their ubiquity and diversity suggests that TA systems may play alternative roles other than protection of mobile DNA [5], [6]. Based on biochemical nature and action mode of antitoxins, five types of TA systems have been proposed to date [7]. The antitoxins of type I or III systems are small RNAs that inhibit toxin expression (type I) or activity (type III). In type II, IV or V TA systems, the antitoxins are low molecular weight proteins which inhibit toxin activity by forming nontoxic TA complexes (type II), shielding of toxin targets (type IV) or specific degradation of toxin mRNAs (type V).

Type II TA systems (hereafter referred to as TA systems) are most prevalent, and are further subdivided into several families according to the molecular identity of toxins [3], [4]. TA toxins exert their effects in different ways. For example, most identified toxins (e. g. RelE, MazF, YafQ, HigB, HicA, MqsR, VapC) are endoribonucleases and inhibit protein synthesis [8]–[13]. TA antitoxins are metabolically unstable due to proteolytic degradation by ATP-dependent proteases Lon and ClpP, and typically consist of a N-terminus DNA-binding domain and a C-terminus toxin-binding domain. The DNA-binding domain, belonging to Helix–Turn–Helix (HTH), Ribbon–Helix–Helix (RHH), AbrB or Phd/YefM class, mediates auto-repression of the TA operon transcription by the antitoxin both alone and in TA complexes, and the toxin-binding domain is responsible for neutralization of the cognate toxin via formation of the TA complex [14]. Interestingly, a particular group of toxins can form TA systems with antitoxins from different protein families [15]. Under favorable growth conditions, the co-expression of an antitoxin in excess of the toxin inhibits the toxin's toxicity through TA complex formation and suppresses the TA operon transcription mediated by the DNA-binding domain present in the antitoxin [14], [16]. When bacteria encounter some circumstances (e.g. amino acid starvation, loss of plasmid) which abolish antitoxin production, degradation by cellular proteases leads to a reduction in antitoxin levels. Consequently, the antitoxin-mediated repression of the TA operon transcription is relieved, and the toxin is released from the TA complex [14], [16]. Thus, both transcriptional and post-transcriptional regulation contribute to diminishing levels of antitoxin and activate the cognate toxin that causes reversible growth inhibition [17] or cell death [18], [19]. This finely tuned regulation of TA systems leads to a proposal that activation of the latent toxin via direct TA complex disruption or some alternative mechanisms may be exploited as a novel bacteria-control strategy [20].

Vap (Virulence associated protein) systems are the largest TA family and are defined by the presence of a PIN-domain protein as the toxic component (VapC) [14], [21]. Yet they are the least well characterized. The PIN domain (PFAM: PF01850) was originally annotated based on sequence similarity to the N-terminal domain of the type IV pili protein, pilT, from *Myxococcus Xanthus* (PIN, PilT N-terminus). Although sharing low sequence similarity, the PIN domain proteins have a highly conserved three-dimensional structure which causes a clustering of four conserved acidic residues to constitute the active site [22]. In VapBC TA systems, the identified toxins show functional conservation in their sequence-specific endoribonuclease activity [23]–[25], and the antitoxins can have any of four DNA-binding motifs found in TA antitoxins [3]. Recently, many genes encoding COG2442 (DUF433) domain proteins of function unknown are found to be associated with PIN family genes, and were proposed to encode VapBC TA systems [3]. However, the COG2442 domain proteins are unrelated to studied TA antitoxins in sequence or secondary structure [3], thus their functional features remain unknown.

The PIN-COG2442 loci are seen in various bacterial genomes but are most abundant in cyanobacteria and chloroflexi. The cyanobacterium *Synechocystis* sp. PCC 6803 (hereafter, *Synechocystis*) encodes at least 16 putative *vapBC* loci on the chromosome [3], [4], as summarized in Table S1 in Supplemental material. Four (*vapBC10*∼*vapBC13*) of those loci encode the COG2442 domain proteins. In this work, we first show that the PIN-COG2442 locus *vapBC10* (*ssr2962/slr1767*) encodes a TA system using *Escherichia coli* which has been successfully used as a host for verification of heterogenic TA modules [26]–[28]. We also demonstrate that the *vapBC10* operon is transcriptionally activated by the antitoxin VapB10 which is degraded by the protease ClpXP2s from *Synechocystis*. This atypical transcription regulation of the *vapBC10* operon is discussed.

## Materials and Methods

### Strains, enzymes and chemicals

*E. coli* strains were grown in LB medium unless otherwise noted. When required, media were supplemented with spectinomycin (100 mg/l) and kanamycin (50 mg/l). All the enzymes including restriction enzymes, ligase, T4 DNA polymerase, T4 polynucleotide kinase and Taq DNA polymerase were purchased from TaKaRa Biotech. PrimeScript 1^st^ strand cDNA synthesis kit was purchased from TaKaRa Biotech as well. [γ-^32^P] ATP was obtained from FuRiDa Biotech. 5′ RACE system for rapid amplification of cDNA ends and Ni-NTA Resin for purification of the expressed proteins were purchased from Invitrogen. Polyclonal goat-anti mouse IgG AP conjugate and chemiluminescence reagent were obtained from Beyotime Biotech. PCR primers are listed in Table S2.

### Plasmid construction

For assays of toxicity, anti-toxicity and growth rescue, selection-expression plasmids were constructed based on the vector pJS298, which contains the arabinose-inducible promoter *P_BAD_* and the isopropyl β-D-thiogalactopyranoside (IPTG)-inducible promoter *P_T7lac_* as well as the genes *araC* and *lacI* encoding the regulator proteins of the respective promoters [29]. The gene *vapB10* was amplified by PCR using the primers slr1767-N and slr1767-K, digested with *Nde*I and *Kpn*I, and cloned behind the promoter *P_T7lac_* of pJS298 generating pJS340. The *vapC10* gene was amplified with the primers ssr2962-S and ssr2962-K, digested with *Sac*I and *Kpn*I, and then placed under the promoter *P_BAD_* of pJS340 obtaining pJS350.

Proteolytic activation plasmids were constructed based on the vectors pJS371 and pJS391 [29], which contain the *Synechosystis* protease genes *lons* and *clPX2s* under control of *P_T7lac_*, respectively. The fragment containing both *vapB10* and *vapC10* was amplified using primers ssr2962-S and slr1767-K, digested with *Sac*I and *Kpn*I, and cloned under *P_BAD_* in pJS371 and pJS391 obtaining pJS427 and pJS429, respectively. The *vapB10* gene was amplified using primers ssr2962-S and ssr2962-K, digested with *Sac*I and *Kpn*I, and sub-cloned under *P_BAD_* in pJS371 and pJS391, producing pJS882 and pJS883, respectively.

For co-expression of *vapB10* with *vapC10*, the fragment containing both *vapB10* and *vapC10* was PCR amplified with the ssr2962-N and slr1767-X. After digested with *Nde*I and *Xho*I, the resulting fragment was cloned into pET30a obtaining pJS653, which allows co-production of VapB10 with the C-terminally hexa-histidine (His_6_)-tagged VapC10 (VapC-His_6_) in *E. coli* BL21(DE3) upon addition of IPTG.

To create *vapBC10*-*lacZ* fusions, we first constructed a reporter vector containing the promoter-less *lacZ* gene of *E. coli*. The 1.2-kb fragment containing a part of the *Synechosystis slr0168*, was PCR generated using primers slr0168-1 and slr0168-2. The PCR product was blunted with T4 DNA polymerase and ligated to the *Pvu*II-digested pUC18 producing pJS378. A *Dra*I fragment containing the Ω cassette, which confers resistance to spectinomycin and streptomycin, from pRL57 [30] was inserted into the blunted *Sal*I site of the *lacZ*-containing plasmid pHB1117 [31], obtaining pJS387. The Ω-*lacZ* fragment was excised with *Pst*I and *XbaI*, blunted and then inserted into the blunted *Eco*RI site of pJS378, obtaining the reporter vector pJS759 (Figure S1). On the basis of vector, we then constructed a series of operon-*lacZ* fusions. The fragment containing the assumed promoter region of *vapBC-10* (*P_vapBC-10_*) was amplified using the primers P_vapBC10_-1 and P_vapBC10_-E2, digested with *Bam*HI and then inserted into the *Bgl*II site of pJS759. The clones with the inserted fragment in the same direction as *lacZ* were identified by PCR analysis with the primers P_vapBC10_-1 and lacZ-R, obtaining the reporter plasmid pJS778 (containing the *P_vapBC10_*-*lacZ* fusion). Similarly, the fragments *P_vapBC10_-vapB10*, *P_vapBC10_*-*vapB10-vapC10* and promoter-less *vapBC10* were generated with the primer pairs P_vapBC10_-1/ssr2962-B2, P_vapBC10_-B1/slr1767-B and ssr2962-B1/slr1767-B, respectively. After *Bam*HI digestion, the fragments were cloned into pJS759, obtaining pJS878 (containing the *P_vapBC10_-vapB10-lacZ* fusion), pJS1028 (containing the *P_vapBC10_-vapB10-vapC10-lacZ* fusion) and pJS1472 (containing the *vapB10-vapC10-lacZ* fusion).

### RNA extraction, RT-PCR reaction and rapid amplification of cDNA ends (RACE)

*Synechocystis* cells were collected from 200 ml culture with an OD_720_ of about 1.0 by centrifugation. The pellet was used for RNA extraction as described previously [32]. Total RNA was converted to cDNA by reverse transcription using PrimeScript 1^st^ strand cDNA synthesis kit according to the manufacturer's instructions. Using 1 µg of cDNA per reaction, the transcript of *vapBC10* was determined by PCR amplification using the primers ssr2962-R and slr1767-R1.

The 5′ end of *vapBC10* transcript was identified using a 5′ RACE system for rapid amplification of cDNA ends kit following the manufacturer's instructions. Briefly, first strand cDNA synthesis was carried out using the total RNA, reverse transcriptase, and the gene specific primer slr1767-R1. The cDNA was purified using the SNAP columns provided in the kit and poly(dC) tails were added to the 3′ends using terminal deoxynucleotidyl transferase. PCR amplification of the tailed cDNA was conducted using the 5′ RACE abridged anchor primer and the first nested primer slr1767-R2. A dilution of the PCR mixture was then subjected to re-amplification using the abridged universal amplification primer with the second nested primer slr1767-R3. The PCR product was sequenced.

### Bacterial growth assays

To investigate toxicity, anti-toxicity and proteolytic activation of VapBC10, drop growth tests were performed, as previously described [33]. For induction of gene expression under control of the promoter *P_T7lac_* or *P_BAD_*, 0.05 mM IPTG or 0.2% (w/v) arabinose was added to M9 medium containing 0.4% (v/v) glycerol (M9+Gly), which served as a single carbon source. Under non-inducing conditions, 0.4% (w/v) glucose instead of 0.4% glycerol was added to M9 medium (M9+Glu), which served as a single carbon source and minimized leaky expression from the *P_T7lac_*/*P_BAD_*-based selection-expression plasmids. *E. coli* BL21(DE3) strains transformed with the respective selection-expression plasmids were grown to exponential phase (an OD_600_ of about 0.6) in LB with 0.4% glucose. Cultures were then spun down, washed twice with liquid M9+Gly medium. The washed cells were resuspended to an OD_600_ of 0.2 in M9+Gly medium, and serially diluted in 10-fold steps. 2 µl of each of diluted samples was dropped on the M9+Glu plates and the M9+Gly plates with IPTG (M9+Gly+IPTG), arabinose (M9+Gly+Ara) as well as IPTG and arabinose (M9+Gly+IPTG+Ara), then incubated at 37°C for 30 h.

For assays of growth rescue, the *E. coli* BL21(DE3) strain containing the selection-expression plasmid pJS350 was grown in LB containing 0.4% glucose. When OD_600_ reached to 0.6, 0.05 mM IPTG was added for induction of *vapC10* expression. At time zero or later time points, aliquots from the IPTG-induced culture were taken, spun down and rinsed twice with M9+Gly medium. The washed cells were resuspended in an equal volume of M9+Gly medium. 100 µl of appropriately diluted samples was spread on the plates M9+Glu (*vapBC10* repressed), M9+Gly+IPTG (*vapC10* continuously induced) and M9+Gly+Ara (*vapB10* induced). The colony-forming unit (CFU) was counted after incubation at 37°C for 30 h.

### Over-expression, purification and antibody preparation of VapB10 and VapC10

The mid-logarithmic-phase culture (OD_600_ of 0.6) of *E. coli* BL21(DE3) containing pJS653 was induced with 1 mM IPTG for 3 h. The cells harvested from the induced culture were sonicated in ice-cold lysis buffer (50 mM NaH_2_PO_4_, 0.3 M NaCl, 10 mM imidazole, 5 mM β-mercaptoethanol, pH 8). Co-purification of the induced proteins from the cleared lysate was conducted by affinity chromatography using Ni-NTA resin under native conditions according to the manufacturer's instructions. Individual purification of VapB10 and VapC10-His_6_ was performed as previously described [17]. Briefly, the cleared lysate was incubated with Ni-NTA agarose and subsequently loaded onto a column. The column was washed extensively in buffer (50 mM NaH_2_PO_4_, 0.3 M NaCl, 35 mM imidazole, 5 mM β-mercaptoethanol, pH 8). The protein VapB10 was eluted from the protein complex with denaturing buffer (100 mM NaH_2_PO_4_, 10 mM Tris-HCl, 8 M Urea, pH 8), and then the protein VapC10-His_6_ was eluted from the column with denaturing elution buffer (100 mM NaH_2_PO_4_, 10 mM Tris-HCl, 8 M Urea, pH 4.5). The purified proteins under denaturing conditions were refolded by a series of sequential dialyses: 1×PBS (pH 8), 0.1% (v/v) TritonX-100, 5 mM DTT; 1×PBS (pH 8), 5 mM DTT; 1×PBS (pH 8), 5 mM DTT and 1×PBS (pH 8), 20% (v/v) glycerol, 5 mM DTT. Concentrations of the purified proteins were determined by the Bradford method.

The recombinant proteins were detected by 18% SDS-PAGE, and the densitometries of the expected protein bands were analyzed using Image J (http://rsb.info.nih.gov/nih-image/). The co-purified proteins were determined by MALDI-TOF mass spectrometry (MS) analysis as described before [33]. Polyclonal antibodies were produced by immunizing New Zealand rabbit with the purified VapB10 and VapC10-His_6_ as previously described [29].

### β-galactosidase activity assay

The *E. coli* DH5α strains containing the corresponding reporter plasmids were grown to an OD_600_ of 0.8, and their β-galactosidase activities were measured and calculated as described previously [34].

### Electrophoretic mobility shift assay (EMSA)

The DNA fragments containing different regions of the promoter *P_vapBC10_* were amplified with the respective primers (Table S1). These DNA fragments were labeled at the 5′ end with [γ-^32^P] ATP using T4 polynucleotide Kinase. For specific and nonspecific binding experiments, the unlabeled fragment (about 1 µM) containing the *P_vapBC10_* or *P_BAD_* region was used as a competitor DNA. Mixtures containing the labeled DNA (about 10 nM) and increasing concentrations of the purified protein were incubated in EMSA buffer (100 mM Tris-HCl, pH 8.0, 100 mM NaCl, 1 mM DTT and 10% glycerol) with 0.1 mg/ml sonicated salmon sperm DNA. Reactions were incubated for 30 min at room temperature, then subjected to 5% native PAGE with 0.5×TBE at 150 V at room temperature for about 1.5 h. Radioactive gels were exposed to a storage phosphor screen, and the images were acquired (GE healthcare).

### Western blot analysis

To detect the stability of VapB10 and VapC10, Western blot experiments were performed using the *E. coli* cells containing the corresponding proteolytic activation plasmids. The cells were grown in the presence of 0.2% arabinose to an OD_600_ of about 0.5, and then 0.1 mM IPTG was added. After further incubation for 30 min (at time zero), 100 mg/ml spectinomycin was supplemented to inhibit protein synthesis. Aliquots from the treated cultures were removed at 20-min intervals for 120 min. Same sample volume was loaded in each well of an SDS-PAGE gel. Protein levels were detected by Western blot analysis using the rabbit polyclonal antibody to VapB10, VapC10, Lons [29] or ClpP2s [29] and a polyclonal goat-anti mouse IgG AP conjugate. The immunoreactive bands on the blot were visualized with enhanced chemiluminescence reagent and exposed to film. Images from exposed film were then analyzed densitometrically using Image J, and the half-lives were calculated using Prism 5 (Graphpad).

## Results

### Promoter characterization and transcription analysis of the *vapBC10* locus

**Figure 1:**
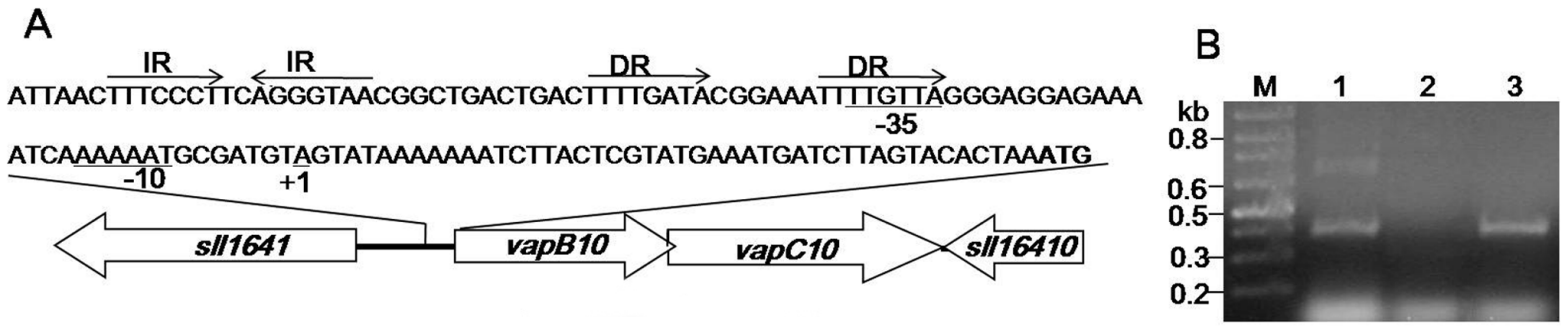
The promoter region and the transcription of the *vapBC10* operon. (A) Schematic representation of the genetic structure of *vapBC10*. DR, direct repeat sequence; IR, inverted repeat sequence; -10, -35 and the transcriptional starting point (TSP) are underlined. (B) Transcription of *vapBC10* determined by RT-PCR analysis using the primers ssr2962-R and slr1767-R1. Lanes: M, molecular weight standard; 1, the genomic DNA; 2, total RNA; 3, cDNA.

To determine the transcriptional start point (TSP) of the promoter *P_vapBC10_*, a 5′-RACE experiment was carried out. Once the TSP was determined, the promoter region was manually checked and was adjusted according to the nucleotide distribution within the *Synechocystis* promoter sequences [35]. Results are summarized in Figure 1A. The *vapBC-10* TSP (+1) is located at an adenine at 45 bp upstream from the ATG codon of *vapB10*. The two conserved regions of the *P_vapBC10_* promoter are TTGTTA and AAAAAT for the -35 and -10 regions, respectively, and are separated by 15 bp. A direct repeat (DR, 5′-TTTTGATA-6N-TTTTGTTA-3′) and an imperfect inverted repeat (IR, 5′-TTTCCCT-2N-AGGGTAA-3′) are found in the promoter region, and located at -62 bp and -28 bp upstream of TSP, respectively. Interestingly, the downstream half of the DR overlaps the deduced -35 hexamer.

To assess whether both *vapB10* and *vapC10* are co-transcribed in *Synechocystis*, a RT-PCR analysis was performed using the primer pair that anneal to the 3′ part of *vapB10* and the 5′ part of *vapC10*. As seen in Figure 1B, a specific amplification product of about 390 bp was observed for the cDNA (lane 3), similar to that of the chromosomal DNA (lane 1). No amplification product was observed for total RNA without reverse transcription (lane 2), eliminating the possibility of DNA contamination. These indicate that both small genes *vapB10* and *vapC10* are co-transcribed, thus forming a bicistronic operon.

### VapC10 inhibits *E. coli* growth which is counteracted by VapB10

**Figure 2:**
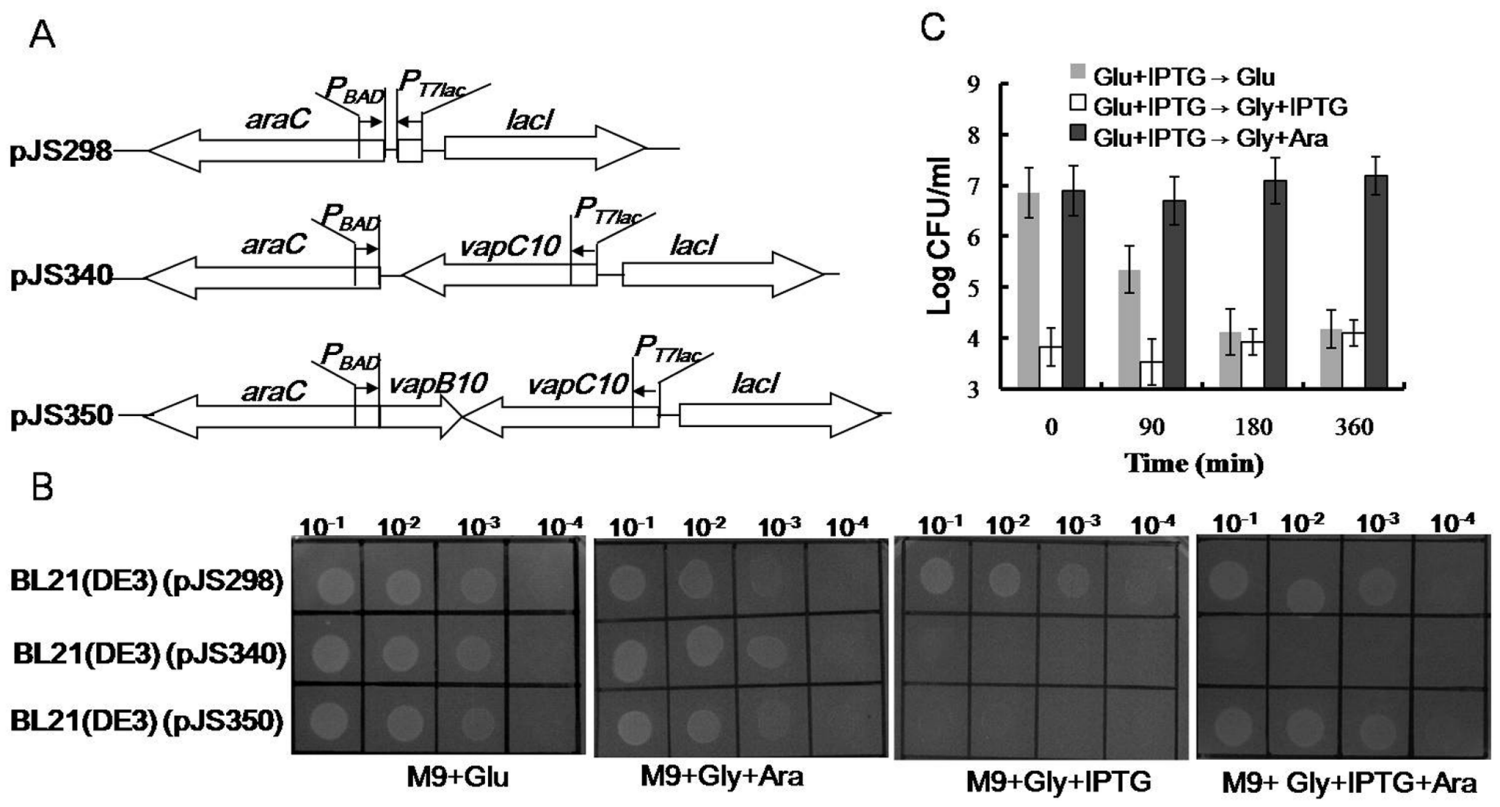
Effects of VapB and/or VapC10 on *E. coli* growth. (A) Schematic diagram showing the structures of the selection expression plasmids. (B) Drop growth experiments of the corresponding selection expression strains. The *E. coli* strain BL21(DE3)(pJS298) was used as the negative control. Diluted samples of each culture were dropped on the indicated M9 plates. (C) CFU counts of the *E. coli* strain BL21(DE3)(pJS350) after the stop of VapC10 production or the subsequent production of VapB10. The IPTG-induced cells of BL21(DE3)(pJS350) were transferred at the indicated time points to the plates M9+Glu (Glu+IPTG→Glu), M9+Gly+IPTG (Glu+IPTG→Gly+IPTG) and M9+Gly+Ara (Glu+IPTG→Gly+Ara). CFUs were counted after incubation at 37°C for 30 h. Error bars indicate the standard errors of the means from three independent experiments.

Although sharing little sequence similarity to the characterized VapC proteins, VapC10 contains a PIN domain with highly conserved quartet of acidic residues at positions 6, 40, 61 and 104 (Figure S2). The secondary structure of VapC10 (Figure S2), predicted with the 3DJIGSAW prediction tool [36] and the DALI server [37], exhibited homology with several well studied VapC toxins, such as the first PIN domain structure for the protein PAE2754 from the archae bacterium *Pyrobaculum aerophilum* (30). To test toxicity and anti-toxicity effects of VapBC10 components, drop growth tests were performed, as described in Materials and methods, using the *E. coli* strains harboring the corresponding selection-expression plasmids (Figure 2A). The strain BL21(DE3)(pJS340) can produce VapC10 upon IPTG induction but does not express VapB10. However, BL21(DE3)(pJS350) can produce VapC10 and/or VapB10 in the presence of IPTG and/or arabinose, respectively. As seen in Figure 2B, all the tested strains showed no difference in drop growth under non-inducing conditions (M9+Glu). However, BL21(DE3)(pJS340) exhibited growth arrest in the presence of IPTG (M9+Gly+IPTG and M9+Gly+IPTG+Ara) but could grow normally in the absence of IPTG (M9+Gly+Ara) compared to the BL21(DE3)(pJS298) control. Also, BL21(DE3)(pJS350) showed growth arrest in the presence of IPTG alone (M9+Gly+IPTG) but could grow under other inducing conditions (M9+Gly+Ara and M9+Gly+IPTG+Ara). Similar growth profiles of these strains were observed in liquid media with the corresponding inducers (data not shown). These indicate that the expression of *vapC10* alone led to growth arrest of *E. coli*, and the simultaneous expression of *vapB10* could neutralize this growth-inhibition effect, suggesting that *vapC10* encodes a TA toxin and *vapB10* encodes the cognate antitoxin.

### VapC10-induced growth arrest can be rescued by the subsequent production of VapB10

To test whether the VapC10-induced growth arrest can be recovered or not, rescue experiments were performed with the strain BL21(DE3)(pJS350). After the culture was induced and treated, as described in Materials and methods, CFUs were enumerated on the plates M9+Glu (*vapBC10* repressed), M9+Gly+IPTG (*vapC10* continuously induced) and M9+Gly+Ara (*vapB10* induced). As seen in Figure 2C, either continuous induction (Glu+IPTG→Gly+IPTG) or stopping induction (Glu+IPTG→Glu, after 180-min induction) of *vapC10* resulted in an about 1000-fold drop in CFU relative to that in which the toxin was not induced (Glu+IPTG→Glu, at time zero). However, no reduction in CFU was observed when *vapB10* was subsequently induced (Glu+IPTG→Gly+Ara). These indicate that the VapC10-induced growth arrest could be overcome by the subsequent expression of VapB10 but not by the stop of VapC10 expression, suggesting a bacteriostatic effect of VapC10.

### Interaction between VapB10 and VapC10

**Figure 3:**
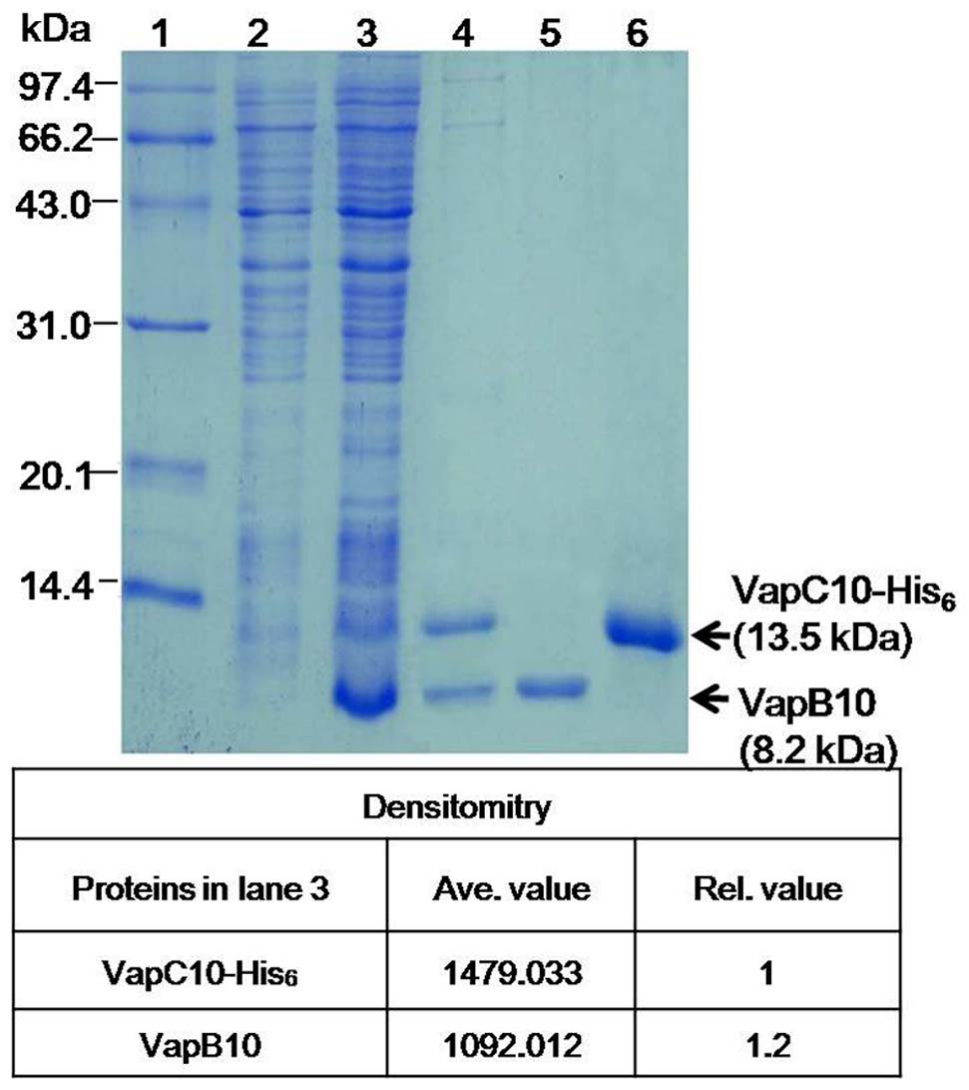
SDS-PAGE analysis of the recombinant proteins from the IPTG-induced cells of *E. coli* BL21(DE3)(pJS653). Lanes: 1, protein molecular weight standard; 2, crude extract of un-induced cells; 3, crude extract of IPTG-induced cells; 4, co-purified proteins under native conditions; 5 and 6, purified and refolded proteins VapB10 and VapC10-His_6_, respectively. The densitometry values and the relative molar ratio of VapB10 to VapC10-His_6_ are shown at the bottom panel.

To test the possible interaction between VapB10 and VapC10, *E. coli* BL21(DE3)(pJS653) was induced with IPTG, and the recombinant proteins were affinity-purified with a Ni-NTA column. As shown in Figure 3, a 8.2-kDa protein and a 13.5-kDa protein were produced after 3 h of induction with IPTG (lane 3), and are consistent with the expected masses of VapB10 and VapC10-His_6_, respectively. A stoichiometry analysis showed that the molar amount of VapB10 exceeded VapC10-His_6_ (Figure 3, lane 3), suggesting that *vapB10* is expressed more effectively than the downstream gene *vapC10* under control of the P*_T7_* promoter in the IPTG-induced cells. In addition, VapB10 was successfully co-purified with VapC-His_6_ under native conditions (Figure 3, lane 4). The MS analyses showed that two VapB10 peaks (836.437, 1584.789 *m*/*z*) and three VapC10-His_6_ peaks (1049.526, 1299.733 and 1455.853 *m*/*z*) were detected in MS-DIGEST program, indicating that both VapB10 and VapC10-His_6_ had the expected peptide masses (Figure S3). These results suggest that VapB10 binds to VapC10-His_6_ forming the TA complex VapBC10 *in vivo*, which may cause the counteraction of the VapC10-induced growth arrest (Figure 2). Besides, the stoichiometry of the Coomassie-stained VapBC10 proteins upon affinity co-purification appears to an approximate 1∶1 ratio of VapB10 to VapC10-His_6_ (Figure 3, lane 4)), indicating that the molar ratio of VapB10 to VapC10-His_6_ is about 1∶1 in the VapBC10 complex. Thus, the co-existence of two different forms of VapB10 bound and unbound to VapC10 during artificial induction *in vivo* is suggested by the observed difference in the molar ratio of VapB10 to VapC10 between the IPTG-induced cells (Figure 3, lane 3) and the affinity-purified VapBC10 complexes (Figure 3, lane 4).

### VapB10 positively auto-regulates the *vapBC10* transcription

**Figure 4:**
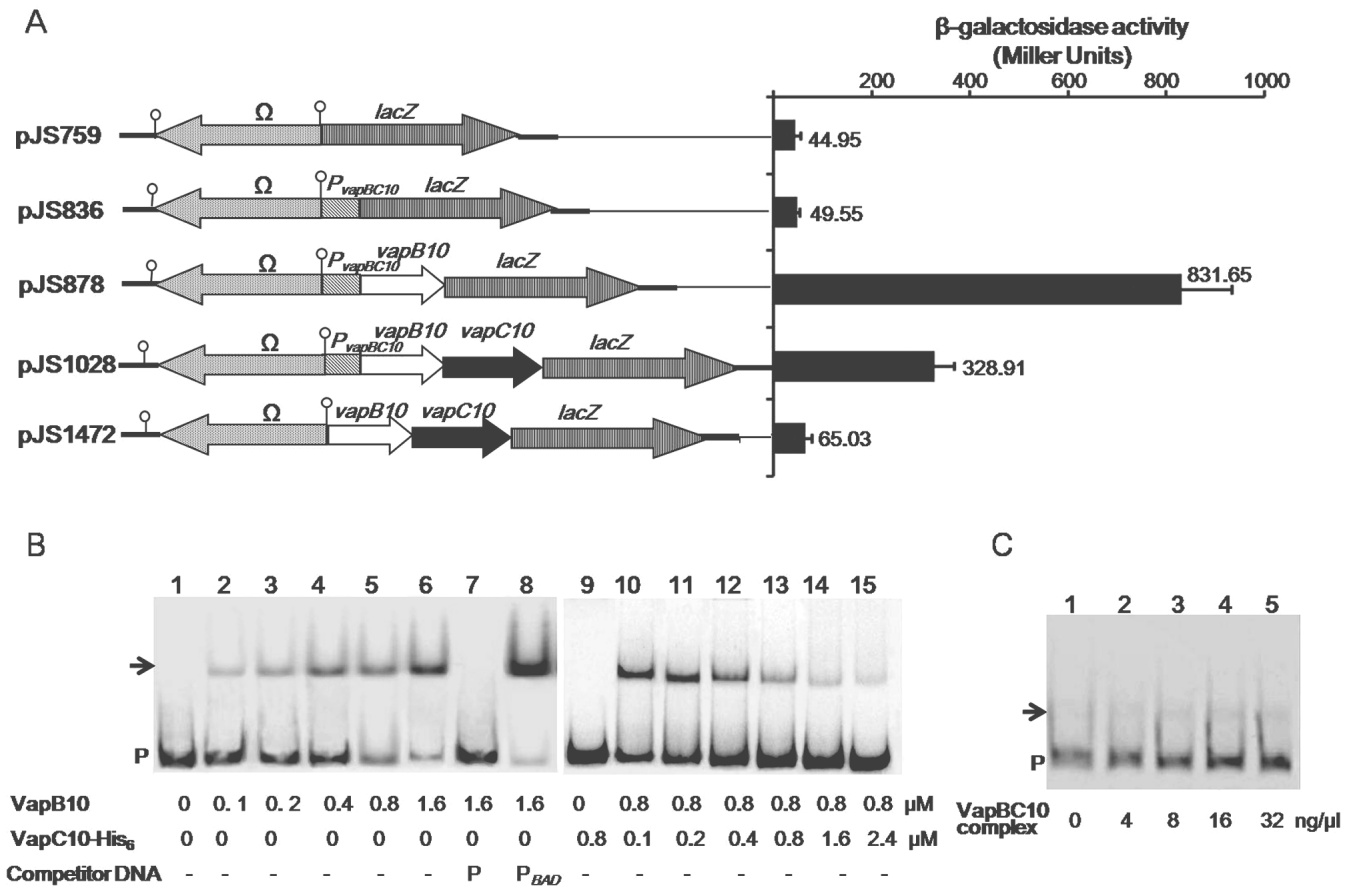
Transcriptional regulation roles and DNA-binding activities of VapBC10 proteins. (A) Effects of VapBC10 components on the transcriptional activity of *P_vapBC10_* measured by β-galactosidase activity assay. The structures of *lacZ* reporter plasmids are shown in the left panel, and the β-galactosidase activities are presented in the right panel. The values are the averages of three independent experiments. Error bars represent standard deviation. (B) EMSAs for the binding of VapBC10 components to the *P_vapBC10_* DNA. A 296-bp DNA fragment P, containing *P_vapBC10_*, was prepared by PCR using the primers P_vapBC10_-E1 and P_vapBC10_-E2. The labeled fragment P was incubated with final concentrations of VapB10 (lanes 1–8) or with VapC10-His_6_ (lanes 9–15) as indicated at the bottom panel. Specific and nonspecific binding events are shown using 1 µM of the unlabeled fragment P (lane 7) or *P_BAD_* (lane 8) obtained from pJS298 by PCR amplification using the primers P_BAD_-F and P_BAD_-R. P indicates unbound DNA and arrows shifted DNA-protein complexes. - symbolizes the absence of the competitor DNA. (C) EMSAs for the binding of the complex VapBC10 to the *P_vapBC10_* DNA. The labeled fragment P was incubated with increasing concentrations of the VapBC10 complex.

It has been demonstrated that TA operons are all negatively auto-regulated at the transcription level by direct binding of antitoxins to the TA operon promoters [14], [16]. To investigate self-regulation of the *vapBC10* transcription, the β-galactosidase activities of the *E. coli* DH5α cells containing the corresponding reporter plasmids (Figure 4A) were measured. The strain which harbors the promoter-less *lacZ* vector pJS759 (Figure 4A) was used as a negative control, and showed a poor β-galactosidase activity. The strain harboring pJS836 that contains the *P_vapBC10_*-*lacZ* fusion had a similar β-galactosidase activity (Figure 4A) with that of the negative control strain. When *vapB10* was introduced, β-galactosidase activity of the strain harboring pJS878 (containing *P_vapBC10_*-*vapB10*-*lacZ* fusion) increased dramatically (Figure 4A). However, the strain carrying pJS1028 (containing *P_vapBC10_*-*vapB10*-*vapC10*-*lacZ* fusion) showed an enzymatic activity significantly lower than that of the strain containing pJS878 but remarkably higher than that of the strain containing pJS836 (Figure 4A). Furthermore, the strain containing pJS1472 (containing *vapB10-vapC10-lacZ* fusion) showed a β-galactosidase activity similar to that of the negative control strain (Figure 4A), eliminating the possibility of a potential promoter present in the *vapBC10* encoding sequence. Therefore, these results suggest that VapB10 is capable of activating the *P_vapBC10_* transcription activity, and the presence of VapC10 partially inhibits this activation effect. Given that the molar ratio of VapB10 to VapC10 produced from the native promoter is similar to that from the IPTG-inducible promoter of pJS653 in *E. coli* (Figure 3), then VapB10 also exists both alone and in the VapBC10 complexes in the *E. coli* strain containing pJS1028 (Figure 4A). Depending on this scenario that may or is expected to occur, one possibility for the transcription inhibition by VapC10 (Figure 4A, as indicated by pJS1028) is that the VapC10-bound VapB10 in the VapBC10 complex fails to activate the *P_vapBC10_* transcription, and only the free VapB10 exerts the effect of transcription activation. Another possibility, not mutually exclusive with the first, is that introduction of the *vapC10* DNA sequence between *P_vapBC10_* and *lacZ* decreases the transcriptional readthrough of the reporter gene. Also, the results of transcription fusion analyses, in agreement with that seen in the RT-PCR analysis (Figure 1B), further indicate that *vapB10* and *vapC10* form a bicistronic operon.

### VapB10 specifically binds the *P_vapBC10_* DNA which is abolished by VapC10

The antitoxin VapB10 belongs to the COG2442 protein family. The structure of one of the COG2442 proteins from *Anabana variabilis* has been solved (PDB:2GA1) by Joint Center for Structural Genomics (JCSG) and has a winged-helix DNA binding domain with a DNA/RNA-binding 3-helical bundle in SCOP (http://scop.mrc-lmb.cam.ac.uk/scop/data/scop.b.c.bgi.b.c.html). Our structure analysis with the 3DJIGSAW prediction tool [36] and the DALI server [37] revealed that VapB10 also contains a DNA/RNA-binding 3-helical bundle fold at its N-terminus (Figure S3), suggesting VapB10 as a DNA-binding antitoxin involved in the auto-regulation of *vapBC10* transcription as seen in Figure 4A.

In order to determine the roles of the VapBC proteins in the observed regulation of *vapBC10* transcription (Figure 4A), EMSAs were performed with the purified VapB10 (Figure 3, lane 5), VapC10-His_6_ (Figure 3, lane 6) or VapBC10 complex (Figure 3, lane 4) as well as the labeled 296-bp DNA DNA fragment P containing the *P_vapBC10_* region. The obtained results revealed that VapB10 was capable of binding to the fragment P (Figure 4B, lane 1–6). In contrast, VapC10 had no DNA-binding activity (Figure 4B, lane 9), and exhibited to neutralize the DNA-binding effect of VapB10 with its increasing amount (Figure 4B, lane 10–15). Furthermore, the purified VapBC10 complexes (Figure 3, lane 4) at the increasing concentrations showed marginal DNA-binding signals (Figure 4C), which may arise from the insignificant amount of VapB10 released from the TA complexes. These indicate that the VapBC10 complex could not bind to the fragment P, and suggest that the transcription-inhibition effect of VapC10 (Figure 4A, indicated as pJS1028) may arise from the inability of the bound VapB10 in the VapBC10 complexes to activate the *P_vapBC10_* activity. Additional control EMSA results showed that, as expected, only the unlabeled fragment P (Figure 4B, lanes 7), and not the unlabeled non-specific fragment P_BAD_ (Figure 4B, lane 8), could competitively inhibit the binding of VapB10 to the labeled fragment P, suggesting a specific physical interaction between VapB10 and the *P_vapBC10_* promoter region. Taken together, these binding results support the notion of a direct regulatory role of VapB10 in *vapBC10* transcription, as suggested by our *lacZ* transcription fusion data (Figure 4A).

### The IR is required for VapB10 binding to the *P_vapBC10_* region

**Figure 5:**
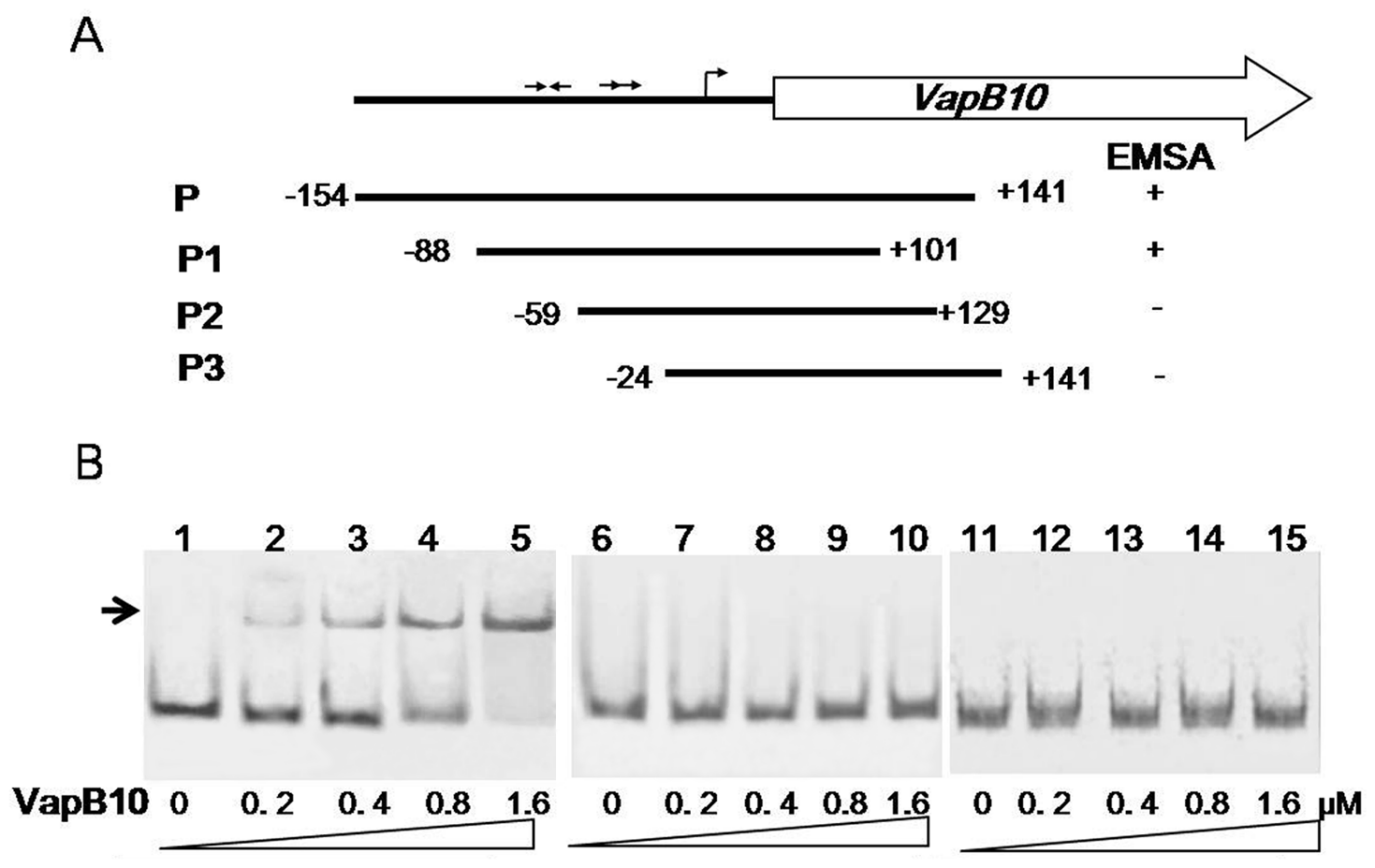
DNA binding regions of VapB10 in the P*_vapBC10_* promoter. (A) Schematic representation of the DNA fragments used in EMSAs. The numbers indicate the ends of the fragments relative to the transcriptional start site. + or − indicates that VapB10 binds to the DNA fragment or not. (B) EMSAs of VapB10 binding to the different regions of the promoter *P_vapBC10_*. The DNA fragments P1, P2 and P3 were PCR amplified with the primer pairs P_vapBC10_-E3/P_vapBC10_-E4, P_vapBC10_-E5/P_vapBC10_-E6 and P_vapBC10_-E7/P_vapBC10_-E2. The labeled fragment P1 (lanes 1-5), P2 (lanes 6-10) and P3 (lanes 11-15) were incubated with increasing concentrations of VapB10 (see the Figure 5 legend for further details), respectively.

In order to identify which sequences within the *P_vapBC10_* promoter region are required for VapB10 binding, three labeled amplicons, namely, P1, P2 and P3, which contain different sections of the promoter region, were generated (Figure 5A). The results obtained from the corresponding EMSAs showed that the VapB10 binding site is localized within a fragment between positions -88 and -59 (Figure 5B). This region contains an imperfect IR (5′-TTTCCCT-2N-AGGGTAA-3′), and does not contain the DR (5′-TTTTGATA-6N-TTTTGTTA-3′), suggesting that the IR plays a role in VapB10 binding.

### ClpXP2s can proteolytically activates VapBC10 in *E. coli*

**Figure 6:**
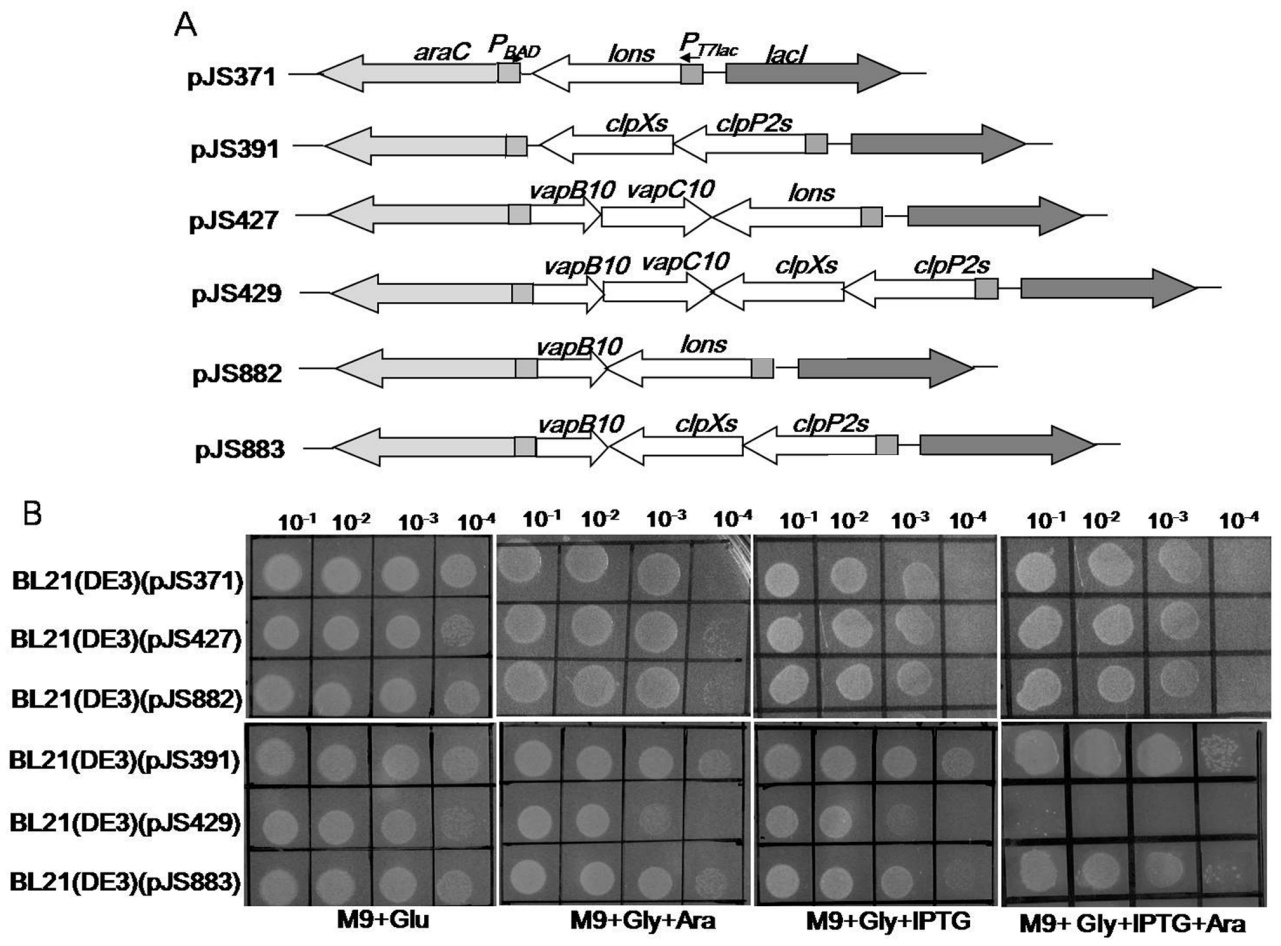
Proteolytic activation effect of the *Synechocystis* protease Lons or ClpXP on VapBC10. (A) Schematic diagram showing the structures of the proteolytic activation plasmids. (B) Drop growth experiments of the corresponding proteolytic activation strains as presented in the right panel. Diluted samples of each culture were dropped on the M9 plates, as described in the Figure 2B legend.

It has been demonstrated that ATP-dependent proteases Lon and ClpP could proteolytically regulate activities of some TA toxins through specific degradation of the antitoxins [9], [14], [38]–[42]. Our previous study showed that both *Synechocystis* proteases Lons and ClpP2s could cleave the RelN antitoxin thus activating RelNE TA system [29]. In order to determine the roles of these two proteases in regulation of the VapC10 toxicity, drop growth experiments were performed using the *E. coli* BL21(DE3) strains containing the proteolytic activation plasmids (Figure 6A). These proteolytic activation strains could conditionally express the *Synechocystis* protease (Lons or ClpXP2s) and/or the VapBC10 components (VapB10 or together with VapC10) upon induction of IPTG and/or arabinose. Because our previous study showed that the growth of the *E. coli* strain containing pJS371 or pJS391 was not affected in the presence of arabinose and/or IPTG [29], here either strain was used as the negative control. As seen in Figure 6B, all the tested strains showed no difference in growth under non-inducing conditions (M9+Glu). However, the strain BL21(DE3)(pJS429) exhibited growth inhibition in the presence of both IPTG and arabinose (M9+Gly+IPTG+Ara) but could grow in the presence IPTG or arabinose (M9+Gly+Ara or M9+Gly+IPTG). Under the same conditions, no difference in drop growth was observed between the other strains tested (Figure 6B). These results indicate that the simultaneous expression of *clpXP2s* along with *vapBC10* caused *E. coli* growth arrest. Since the production of VapC10 caused *E. coli* growth arrest in the absence of VapB10 (Figure 2B and C), we speculated that ClpXP2s, rather than Lons, may activate VapC10 via specific proteolysis of VapB10, allowing VapC10 to be released from the VapBC10 complexes.

### VapB10 is specifically degraded by ClpXP2s

**Figure 7:**
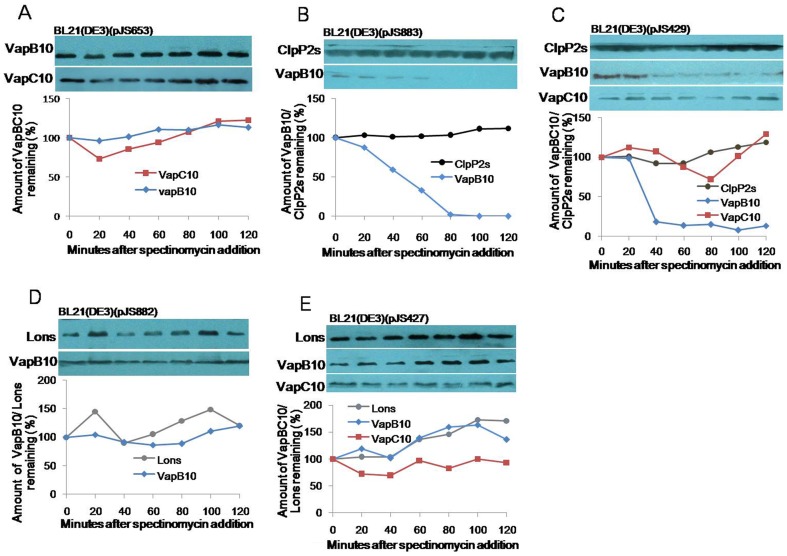
Stability of VapB10 and VapC10 in the presence of ClpPXP2s or Lons. The *E. coli* strains BL21(DE3)(pJS653) (A), BL21(DE3)(pJS883) (B), BL21(DE3)(pJS429) (C), BL21(DE3)(pJS882) (D) and BL21(DE3)(pJS427) (E) were grown, induced and translationally stalled as described in Materials and methods. The cells treated at various points of time were subjected to Western blot analysis to monitor VapB10, VapC10, ClpP2s or Lons with the respective primary antibodies. The corresponding graph represents the percentages of the related protein amount at each time point compared to that at time zero.

To determine the possible proteolytic degradation, we investigated the stability of VapBC10 proteins in the presence of ClpXP2s or Lons. The strains containing the corresponding proteolytic activation plasmids were grown and treated, as described in Materials and methods, and the treaded cells were subjected to Western blot analysis to monitor VapB10, VapC10, ClpP2s or Lons with the respective primary antibodies. Based on densitometrical analyses of Western blots, the percentage of the related protein amount at each time point compared to that at time zero was calculated, and the half-lives of proteins were determined. We first probed the proteolysis effects of *E. coli* proteases on VapB10 and VapC10 using the strain *E. coli* BL21(DE3)(pJS653) (for co-expression of VapB10 with VapC10 in Figure 3). The results revealed that the levels of VapB10 and VapC10 remained unaltered during translation stall elicited by spectinomycin addition, indicating that the *E. coli* proteases degraded neither VapB10 nor VapC10. To test the role of ClpXP2s in the proteolysis of VapBC10 proteins, the stability of VapB10 or with VapC10 was investigated in the strain BL21(DE3)(pJS883) or BL21(DE3)(pJS429). The results showed that ClpP2s remained stable in the BL21(DE3)(pJS883) cells during translation inhibition, while the VapB10 level rapidly decreased with a half-life of about 40 min (Figure 7B). In the BL21(DE3)(pJS429) cells, the levels of ClpP2s and VapC10 remained unchanged over a 2-hour period of translation arrest, but the VapB10 level showed to be decreased with a half-life (Figure 7C) similar to that observed in the strain BL21(DE3)(pJS883) (Figure 7B). These indicate that ClpXP2s could degrade VapB10 regardless of the presence or absence of VapC10. As suggested by the result of drop growth experiments (Figure 6B), ClpXP2s could degrade VapB10 in the TA complexes and consequently activate the latent toxicity of the VapBC10 system. When we determined the proteolysis role of Lons in VapBC10 proteins by Western blot analysis using the strains BL21(DE3)(pJS882) and BL21(DE3)(pJS427), the levels of VapB10 and VapC10 remained stable over the course of translation inhibition (Figure 7D and E). Thus, Lon could not degrade VapBC10 proteins, consistent with our drop growth evidence (Figure 6B).

## Discussion

An important step toward understanding the function of VapBC TA systems is the elucidation of their features. We here characterized the *Synechocystis* chromosomal PIN-COG2442 locus *vapBC10*. The *vapB10* gene was transcriptionally coupled with the *vapC10* gene, forming a bicistronic operon (Figure 1 and 4). The production of the PIN-domain protein VapC10 inhibited *E. coli* growth, which could be overcome by the simultaneous or subsequent production of the COG2442 domain protein VapB10 *in trans* (Figure 2) through formation of the protein-protein complex (Figure 3). These suggest that the *vapBC10* operon encodes a VapBC TA system. It has been demonstrated that the characterized VapC toxins share conservation of function in their ribonuclease activity although little sequence homology. For example, the *Shigella* and *Salmonella* VapC toxins were shown to cleave fMet tRNA at a single site between the anti-codon stem and loop [23]. The *Mycobacterium tuberculosis* VapC (Rv0595c) also exhibited comparatively weak RNA endoribonuclease activity [25]. Recently, the sequence-specific ribonuclease activities of four VapC proteins were successfully determined from two different organisms *Pyrobaculum aerophilum* (PAE0151 and PAE2754) and *M. tuberculosis* (Rv0065 and Rv0617) [24]. Therefore, it is conceivable that VapC10 may exert its growth-arrest effect via translation inhibition similar to the identified VapC toxins although its biochemical mechanism of action still remains to be determined.

Generally, TA operon transcription is auto-repressed by the antitoxin both alone and in TA complexes, and toxin activity is regulated by proteases Lon and ClpP [14], [16]. It is proposed that normal growth conditions allow to inhibit toxin activity by nontoxic TA complex formation which also represses TA expression, while particular circumstances cause derepression of TA expression and activation of toxins due to antitoxin proteolysis by proteases [14], [16]. Interestingly, our results from both *lacZ* transcription fusion analysis in *E. coli* and EMSA indicate that VapB10 positively auto-regulated *vapBC10* expression via its binding to the *P_vapBC10_* region that includes an IR that is a strong candidate for recognition by the protein, while the TA complex VapBC10 exhibited no regulation activity due to its inability to bind the promoter *P_vapBC10_* (Figure 4 and 5). These suggest that the *vapBC10* operon may possess a regulatory mechanism different from those of the characterized TA systems. However, because of possible involvement of specific host factors that might substantially change the regulatory pattern observed here, an exact mechanism of transcriptional regulation of the *vapBC10* operon remains to be elucidated in the native host *Synechocystis*. Nevertheless, our characterization of the transcriptional auto-regulation of the *vapBC10* operon here provides important information for further research directions.

A recent study suggests a new paradigm for regulation of TA operon transcription by a specific host factor [43]. In Nontypeable *Haemophilus influenza* (NTHi), Fis (a global trans-activator factor for inversion stimulation) stimulates the *vapBC-1* operon expression on growth resumption. The TA complex VapBC-1 can transcriptionally suppress its own operon when *fis* expression drops in early log phase growth. However, VapBC-1 auto-regulates its own operon through VapC-1 binding to DNA, with VapB1 targeting the TA complex to the translation initiation region (TIR) of *vapB-1*. During nutrient deprivation or environmental stress, VapB-1 would be degraded by proteases Lon and Clp, freeing VapC-1 from nontoxic VapBC-1 complexes [14]. Since the VapC-1 interaction with vapB-1 TIR appears less stable in the absence of VapB-1, it may be readily displaced by Fis-induced structural changes in the promoter when conditions favor the resumption of bacterial growth. Based on this unusual mode for auto-regulation of TA operon transcription, it is tempting to speculate that other transcriptional factor(s) in *Synechocystis*, similar to the NTHi Fis, may be involved in the regulation of *vapBC10* transcription under certain growth conditions. In addition, our sequence analysis of the *Syenchocystis* VapBC TA systems (Table S1) revealed that the components of the four PIN-COG2442 VapBC systems share the most homology relative to those of the others in *Synechocystis*. VapC10 has 24.1% and 17.1% sequence identity to VapC12 and VapC13, and VapB10 has 28.4%, 41.9% and 31.1% identity to VapB11, VapB12 and VapB13, respectively. The occurrence in *Synechocystis* of multiple COG2442-PIN VapBC components that are related in sequence raises the possibility that interactions occur between noncognate pairs. Such noncognate interactions may also be involved in regulation of *vapBC10* transcription and VapC10 activity. Our experiments are underway to determine the affinity between noncognate VapBC protein pairs.

Another concern is the *Synechocystis* proteases because ATP-dependant proteases generally regulated both TA operon transcription and toxin activity via antitoxin proteolysis. Compared to other bacteria, cyanobacteria have quite complex protease system which remains largely unexplained. In *Synechocystis*, the genes *slr0542*, *sllo534*, *slr0165* and *slr0164* are predicted to encode four distinct proteolytic subunits ClpP1s, ClpP2s, ClpP3s and ClpRs, and *sll0020*, *sll0535*, *slr0156* and *slr1641* encode four ClpP ATPase-chaperones ClpCs, ClpXs, ClpB1s and ClpB2s [44]. The gene *clpP2s* (*sll0534*) overlaps with *clpXs* (*sll0535*) by 3 nucleotides. Our sequence analysis showed that ClpP2s is homologous (ca. 61.4% sequence identity) to the single ClpP of *E*. *coli*, and ClpXs has 64.1% sequence identity to the *E. coli* ClpX. The gene *slr0195* encodes the *E. coli* Lon homolog Lons (ca 20.7% sequence identity). These suggest that the *Synechocystis* proteases Lons and ClpP2s may be responsible for proteolysis of TA antitoxins, as their homologs described in other bacteria [9], [14], [38]–[42]. Our studies also demonstrated that both proteases Lons and ClpP2s could degrade the antitoxin RelN of the RelNE TA system [29], while ClpXP2s, rather than Lon, proteolytically regulate the VapC10 activity by cleaving VapB10 (Figure 6 and 7). Neverthless, the possible involvement of other *Synechocystis* ClpP proteases in VapB10 proteolysis and their physiological roles remain under further investigation. The cyanobacterium *Synechococcus elongate* possesses a *clp* gene set and organization similar to those of *Synechocystis*. In this strain, *clpP2* inactivation does not cause obvious phenotypic changes under the conditions tested [45], while loss of ClpP1 significantly affects the ability of *Synechococcus* to acclimate to some adverse growth conditions [46], [47]. Either *clpR* or *clpP3* is essential for cell viability, and thus cannot be deleted [48], [49]. Unfortunately, our repeated attempts to obtain a *Synechocystis* mutant with complete deletion of *clpP2s* have been unsuccessful, suggesting different physiological roles of ClpP homologs in various cyanobacteria species.

Exploiting the properties of TA systems has led to a number of applications for various purposes. For example, the finely tuned regulation of inherent anti-bacterial activity of TA toxins could be employed to inhibit bacterial growth by artificial activation of TA systems [20]. As a novel anti-bacterial strategy, TA toxins could be activated by preventing or disrupting TA complex formation. One of the prerequisites for successful application of this strategy is to select functional TA systems suitable for artificial activator. If the TA system VapBC10 shares the properties, especially the potential toxicity and the positive auto-regulation, in its native host *Synechocystis* as those here seen in *E. coli*, it may be a desirable target for study of TA system-mediated anti-bacteria strategy. Thus, characterization of such TA systems in cyanobacteria represents a promising avenue for developing novel and effective strategies for cyanobacteria-bloom control.

## Supporting Information

Figure S1 **The schematic representation of the reporter vector pJS759.** The start codon of the promoter-less *lacZ* is in bold face. The ribosome binding site (RBS) is underlined. The boxed triad bases are the three stop codons in different potential reading frames to keep from a translational fusion with the *lacZ* reporter gene. The stem-loop symbols at both ends of the Ω cassette indicate the short inverted repeats that terminate background transcription.
(TIF)

Figure S2 **Sequences and structures of VapB10 and VapC10.** Shown are the amino acid sequences of VapC10 and VapB10 with their secondary structure elements assigned according to structure analysis with the 3DJIGSAW prediction tool and the DALI server. Putative catalytic residues of VapV10 are marked with a star.
(TIF)

Figure S3 **Identification of VapB10 and VapC10-His_6_ by MS analysis**. Both VapB10 (A) and VapC10-His_6_ (B) from lane 4 in the Figure 3 were confirmed by MS analysis. Shown below are amino acid sequences and predicted m/z values of VapB10 and VapC10 by online analysis using the MS-DIGEST program (http://prospector.ucsf.edu).
(TIF)

Table S1 The putative *vapBC* TA loci on the *Synechoycystis* chromosome.
(DOCX)

Table S2 The PCR primers used in this study.
(DOCX)

## References

[1] Jaffe A, Ogura T, Hiraga S (1985) Effects of the *ccd* function of the F plasmid on bacterial growth. J Bacteriol 163: 841–849. 3897195 10.1128/jb.163.3.841-849.1985 PMC219208

[2] Pandey DP, Gerdes K (2005) Toxin-antitoxin loci are highly abundant in free-living but lost from host-associated prokaryotes. Nucleic Acids Res 33: 966–976. 15718296 10.1093/nar/gki201 PMC549392

[3] Makarova K, Wolf Y, Koonin E (2009) Comprehensive comparative-genomic analysis of Type 2 toxin-antitoxin systems and related mobile stress response systems in prokaryotes. Biol Direct 4 : doi:10.1186/1745-6150-1184-1119. 10.1186/1745-6150-4-19 PMC2701414 19493340

[4] Leplae R, Geeraerts D, Hallez R, Guglielmini J, Dreze P, et al (2011) Diversity of bacterial type II toxin-antitoxin systems: a comprehensive search and functional analysis of novel families. Nucleic Acids Res 39: 5513–5525. 21422074 10.1093/nar/gkr131 PMC3141249

[5] Magnuson RD (2007) Hypothetical functions of toxin-antitoxin systems. J Bacteriol 189: 6089–6092. 17616596 10.1128/JB.00958-07 PMC1951896

[6] Van Melderen L, Saavedra De Bast M (2009) Bacterial toxin-antitoxin systems: more than selfish entities? PLoS Genet 5: e1000437. 19325885 10.1371/journal.pgen.1000437 PMC2654758

[7] Schuster CF, Bertram R (2013) Toxin-antitoxin systems are ubiquitous and versatile modulators of prokaryotic cell fate. FEMS Microbiol Lett 340: 73–85. 23289536 10.1111/1574-6968.12074

[8] Yamaguchi Y, Park JH, Inouye M (2009) MqsR, a crucial regulator for quorum sensing and biofilm formation, is a GCU-specific mRNA interferase in *Escherichia coli* . J Biol Chem 284: 28746–28753. 19690171 10.1074/jbc.M109.032904 PMC2781420

[9] Christensen SK, Pedersen K, Hansen FG, Gerdes K (2003) Toxin-antitoxin loci as stress-response-elements: ChpAK/MazF and ChpBK cleave translated RNAs and are counteracted by tmRNA. J Mol Biol 332: 809–819. 12972253 10.1016/s0022-2836(03)00922-7

[10] Christensen-Dalsgaard M, Gerdes K (2006) Two *higBA* loci in the *Vibrio cholerae* superintegron encode mRNA cleaving enzymes and can stabilize plasmids. Mol Microbiol 62: 397–411. 17020579 10.1111/j.1365-2958.2006.05385.x

[11] Jørgensen MG, Pandey DP, Jaskolska M, Gerdes K (2009) HicA of *Escherichia coli* defines a novel family of translation-independent mRNA interferases in bacteria and archaea. J Bacteriol 191: 1191–1199. 19060138 10.1128/JB.01013-08 PMC2631989

[12] Pedersen K, Zavialov AV, Pavlov MY, Elf J, Gerdes K, et al (2003) The bacterial toxin RelE displays codon-specific cleavage of mRNAs in the ribosomal A site. Cell 112: 131–140. 12526800 10.1016/s0092-8674(02)01248-5

[13] Prysak MH, Mozdzierz CJ, Cook AM, Zhu L, Zhang Y, et al (2009) Bacterial toxin YafQ is an endoribonuclease that associates with the ribosome and blocks translation elongation through sequence-specific and frame-dependent mRNA cleavage. Mol Microbiol 71: 1071–1087. 19210620 10.1111/j.1365-2958.2008.06572.x

[14] Gerdes K, Christensen SK, Lobner-Olesen A (2005) Prokaryotic toxin-antitoxin stress response *loci* . Nat Rev Microbiol 3: 371–382. 15864262 10.1038/nrmicro1147

[15] Anantharaman V, Aravind L (2003) New connections in the prokaryotic toxin-antitoxin network: relationship with the eukaryotic nonsense-mediated RNA decay system. Genome Biol 4: R81. 14659018 10.1186/gb-2003-4-12-r81 PMC329420

[16] Yamaguchi Y, Inouye M (2011) Regulation of growth and death in *Escherichia coli* by toxin-antitoxin systems. Nat Rev Microbiol 9: 779–790. 21927020 10.1038/nrmicro2651

[17] Pedersen K, Christensen S, Gerdes K (2002) Rapid induction and reversal of a bacteriostatic condition by controlled expression of toxins and antitoxins. Mol Microbiol 45: 501–510. 12123459 10.1046/j.1365-2958.2002.03027.x

[18] Amitai S, Yassin Y, Engelberg-Kulka H (2004) MazF-mediated cell death in *Escherichia coli*: a point of no return. J Bacteriol 186: 8295–8300. 15576778 10.1128/JB.186.24.8295-8300.2004 PMC532418

[19] Nariya H, Inouye M (2008) MazF, an mRNA interferase, mediates programmed cell death during multicellular *Myxococcus* development. Cell 132: 55–66. 18191220 10.1016/j.cell.2007.11.044

[20] Williams JJ, Hergenrother PJ (2012) Artificial activation of toxin-antitoxin systems as an antibacterial strategy. Trends Microbiol 20: 291–298. 22445361 10.1016/j.tim.2012.02.005 PMC3952271

[21] Arcus VL, McKenzie JL, Robson J, Cook GM (2011) The PIN-domain ribonucleases and the prokaryotic VapBC toxin-antitoxin array. Protein Eng Des Sel 24: 33–40. 21036780 10.1093/protein/gzq081

[22] Arcus VL, McKenzie JL, Robson J, Cook GM (2011) The PIN-domain ribonucleases and the prokaryotic VapBC toxin-antitoxin array. Protein Eng Des Sel 24: 33–40. 21036780 10.1093/protein/gzq081

[23] Winther KS, Gerdes K (2011) Enteric virulence associated protein VapC inhibits translation by cleavage of initiator tRNA. Proc Natl Acad Sci U S A 108: 7403–7407. 21502523 10.1073/pnas.1019587108 PMC3088637

[24] McKenzie JL, Duyvestyn JM, Smith T, Bendak K, MacKay J, et al (2012) Determination of ribonuclease sequence-specificity using Pentaprobes and mass spectrometry. RNA 18: 1267–1278. 22539524 10.1261/rna.031229.111 PMC3358648

[25] Sharp JD, Cruz JW, Raman S, Inouye M, Husson RN, et al (2012) Growth andtranslation inhibition through sequence-specific RNA binding by *Mycobacterium tuberculosis* VapC Toxin. J Biol Chem 287: 12835–12847. 22354968 10.1074/jbc.M112.340109 PMC3339977

[26] Florek P, Muchova K, Pavelcikova P, Barak I (2008) Expression of functional *Bacillus* SpoIISAB toxin-antitoxin modules in *Escherichia coli* . FEMS Microbiol Lett 278: 177–184. 18096016 10.1111/j.1574-6968.2007.00984.x

[27] Zhao LX, Zhang JJ (2008) Biochemical characterization of a chromosomal toxin-antitoxin system in *Mycobacterium tuberculosis* . FEBS Lett 582: 710–714. 18258191 10.1016/j.febslet.2008.01.045

[28] Syed MA, Koyanagi S, Sharma E, Jobin MC, Yakunin AF, et al (2011) The chromosomal *mazEF* Locus of *Streptococcus mutans* encodes a functional type II toxin-antitoxin addiction system. J Bacteriol 193: 1122–1130. 21183668 10.1128/JB.01114-10 PMC3067577

[29] Ning D, Ye S, Liu B, Chang J (2011) The proteolytic activation of the *relNEs* (*ssr1114/slr0664*) toxin-antitoxin system by both proteases Lons and ClpP2s/Xs of *Synechocystis* sp. PCC 6803. Curr Microbiol 63: 496–502. 21909782 10.1007/s00284-011-0011-5

[30] Cai Y, Wolk CP (1990) Use of a conditionally lethal gene in *Anabaena* sp. strain PCC 7120 to select for double recombinants and to entrap insertions. J Bacteriol 172: 3138–3145. 2160938 10.1128/jb.172.6.3138-3145.1990 PMC209118

[31] Gao H, Xu X (2007) Construction of copper induced gene expression platform in *Synechosystis* sp. PCC6803. Acta Hydrobiologica Sinica 31: 240–244.

[32] Ning D, Xu X (2004) *alr0117*, a two-component histidine kinase gene, is involved in heterocyst development in *Anabaena* sp. PCC 7120. Microbiology 150: 447–453. 14766923 10.1099/mic.0.26747-0

[33] Ning DG, Jiang Y, Liu ZY, Xu QG (2013) Characterization of a chromosomal type II toxin-Antitoxin system *mazEaFa* in the cyanobacterium *Anabaena* sp PCC 7120. PLoS One 8(2): e56035. 23451033 10.1371/journal.pone.0056035 PMC3581536

[34] Miller JH (1972) Experiments in molecular genetics. Cold Spring Harbor Laboratory Pr. pp. 352–355.

[35] Mitschke J, Georg J, Scholz I, Sharma CM, Dienst D, et al (2011) An experimentally anchored map of transcriptional start sites in the model cyanobacterium *Synechocystis* sp PCC6803. Proc Natl Acad Sci U S A 108: 2124–2129. 21245330 10.1073/pnas.1015154108 PMC3033270

[36] Bates PA, Kelley LA, MacCallum RM, MJE S (2001) Enhancement of protein modelling by human intervention in applying the automatic programs 3D-JIGSAW and 3D-PSSM. Protein Struct Funct Genet (Suppl) 5: 39–46. 10.1002/prot.1168 11835480

[37] Holm L, Rosenstrom P (2010) Dali server: conservation mapping in 3D. Nucleic Acids Res 38: W545–549. 20457744 10.1093/nar/gkq366 PMC2896194

[38] Christensen SK, Mikkelsen M, Pedersen K, Gerdes K (2001) RelE, a global inhibitor of translation, is activated during nutritional stress. Proc Natl Acad Sci U S A 98: 14328–14333. 11717402 10.1073/pnas.251327898 PMC64681

[39] Roberts RC, Ström AR, Helinski DR (1994) The *parDE* operon of the broad-host-range plasmid RK2 specifies growth inhibition associated with plasmid loss. J Mol Biol 237: 35–51. 8133518 10.1006/jmbi.1994.1207

[40] Hilliard JJ, Simon LD, Van Melderen L, Maurizi MR (1998) PinA inhibits ATP hydrolysis and energy-dependent protein degradation by Lon protease. J Biol Chem 273: 524–527. 9417111 10.1074/jbc.273.1.524

[41] Cherny I, Gazit E (2004) The YefM antitoxin defines a family of natively unfolded proteins: implications as a novel antibacterial target. J Biol Chem 279: 8252–8261. 14672926 10.1074/jbc.M308263200

[42] Donegan NP, Thompson ET, Fu Z, Cheung AL (2010) Proteolytic regulation of toxin-antitoxin systems by ClpPC in *Staphylococcus aureus* . J Bacteriol 192: 1416–1422. 20038589 10.1128/JB.00233-09 PMC2820870

[43] Cline SD, Saleem S, Daines DA (2012) Regulation of the vapBC-1 Toxin-Antitoxin Locus in Nontypeable *Haemophilus influenzae* . PLoS One 7(3): e32199. 22427824 10.1371/journal.pone.0032199 PMC3302801

[44] Kaneko T, Sato S, Kotani H, Tanaka A, Asamizu E, et al (1996) Sequence analysis of the genome of the unicellular cyanobacterium *Synechocystis* sp. strain PCC6803. II. Sequence determination of the entire genome and assignment of potential protein-coding regions. DNA Res 3: 109–136. 8905231 10.1093/dnares/3.3.109

[45] Schelin J, Lindmark F, Clarke A K (2002) The *clpP* multigene family for the ATPdependent Clp protease in the cyanobacterium *Synechococcus* . Microbiology 14: 2255–2265. 10.1099/00221287-148-7-2255 12101312

[46] Clarke AK, Schelin J, Porankiewicz J (1998) Inactivation of the clpPI gene for the proteolytic subunit of the ATP-dependent Clp protease in the cyanobacterium *Synechococcus* limits growth and light acclimation. Plant Mol Biol 37: 791–801. 9678574 10.1023/a:1006016302074

[47] Porankiewicz J, Schelin J, Clarke AK (1998) The ATP-dependent Clp protease is essential for acclimation to UV-B and low temperature in the cyanobacterium *Synechococcus* . Mol Microbiol 29: 275–283. 9701820 10.1046/j.1365-2958.1998.00928.x

[48] Schelin J, Lindmark F, Clarke AK (2002) The clpP multigene family for the ATP-dependent Clp protease in the cyanobacterium *Synechococcus* . Microbiology 148: 2255–2265. 12101312 10.1099/00221287-148-7-2255

[49] Clarke AK, Eriksson MJ (1996) The cyanobacterium *Synechococcus* sp. PCC 7942 possesses a close homologue to the chloroplast ClpC protein of higher plants. Plant Mol Biol 31: 721–730. 8806403 10.1007/BF00019460

