# Supplementary figures and images for: Transcriptional and Proteolytic Regulation of the Toxin-Antitoxin Locus vapBC10 (ssr2962/slr1767) on the Chromosome of Synechocystis sp. PCC 6803
Source: PLoS One. 2013 Nov 19;8(11):e80716. doi: 10.1371/journal.pone.0080716 (PMC3834315; doi:10.1371/journal.pone.0080716)

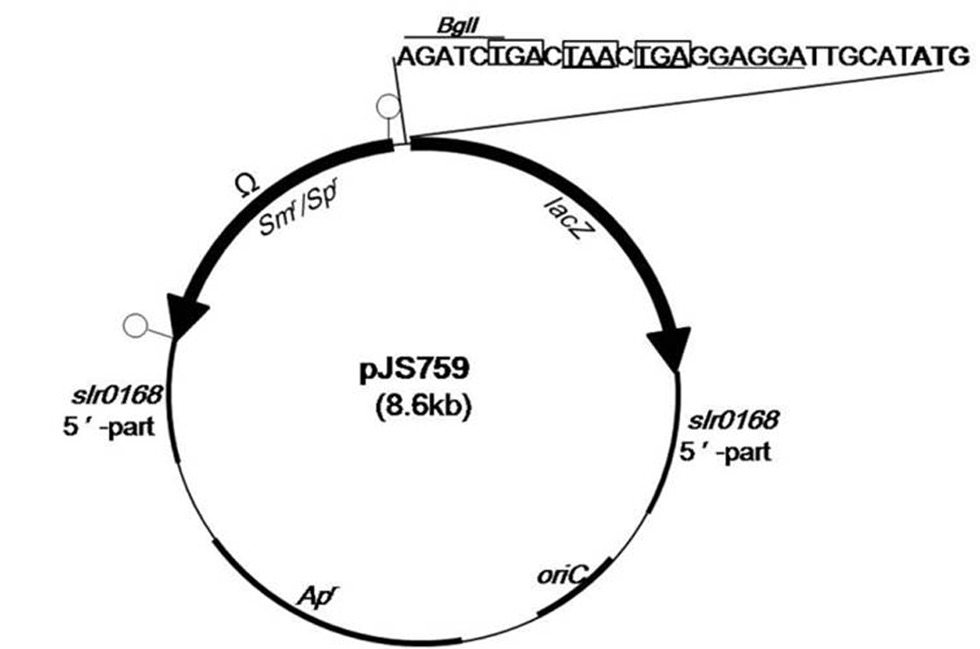

Supplement: Figure S1 — The schematic representation of the reporter vector pJS759. The start codon of the promoter-less lacZ is in bold face. The ribosome binding site (RBS) is underlined. The boxed triad bases are the three stop codons in different potential reading frames to keep from a translational fusion with the lacZ reporter gene. The stem-loop symbols at both ends of the Ω cassette indicate the short inverted repeats that terminate background transcription. (TIF) [file pone.0080716.s001.tif]

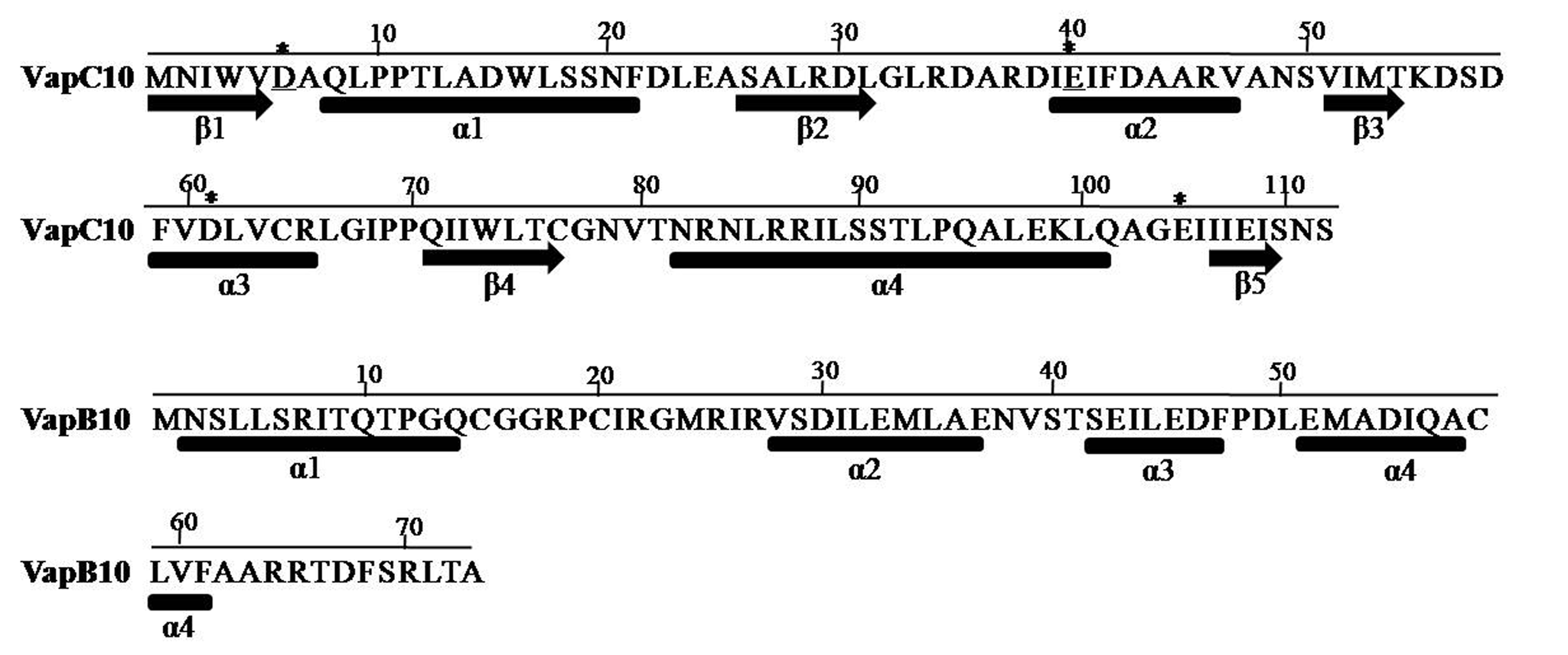

Supplement: Figure S2 — Sequences and structures of VapB10 and VapC10. Shown are the amino acid sequences of VapC10 and VapB10 with their secondary structure elements assigned according to structure analysis with the 3DJIGSAW prediction tool and the DALI server. Putative catalytic residues of VapV10 are marked with a star. (TIF) [file pone.0080716.s002.tif]

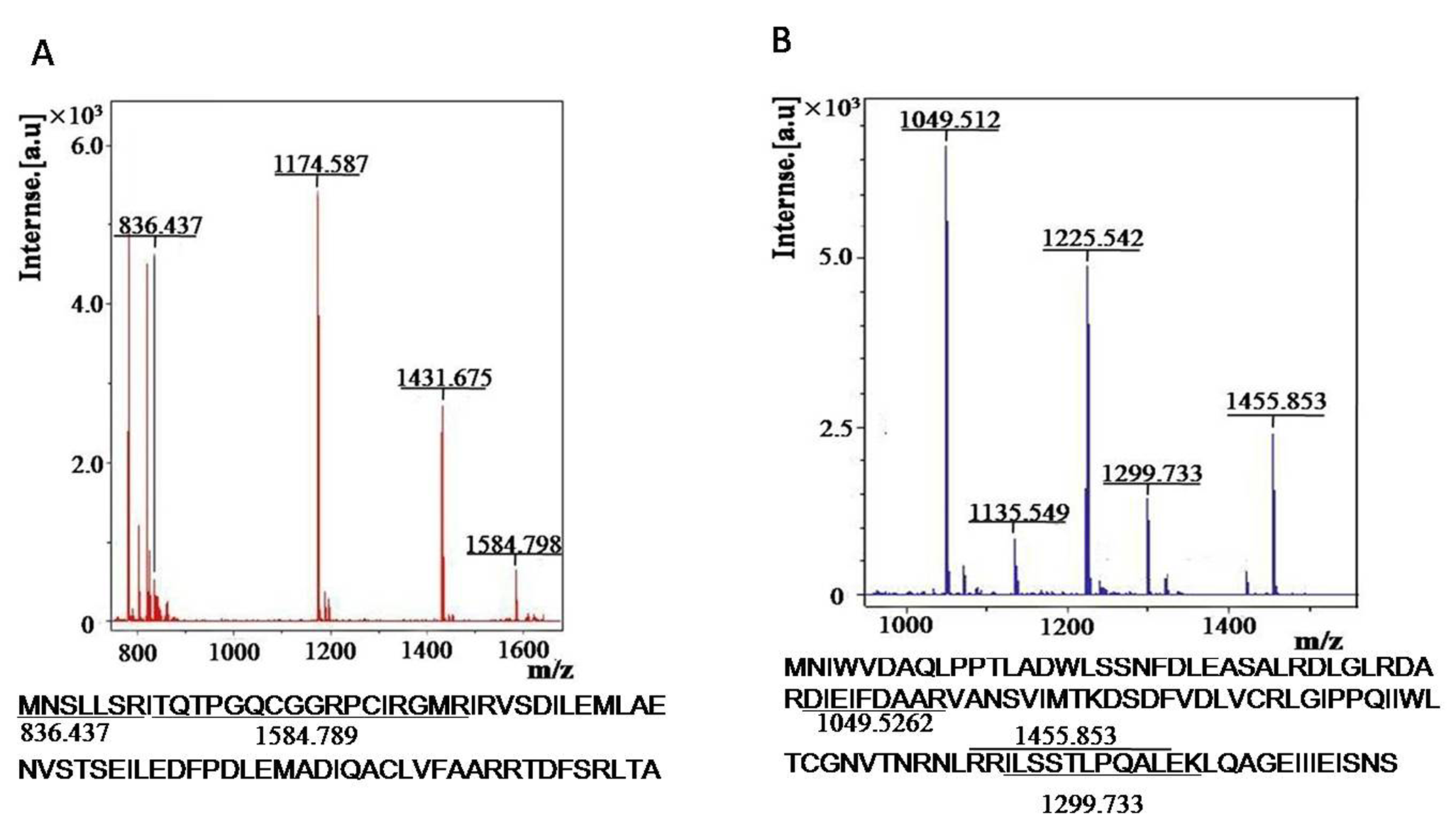

Supplement: Figure S3 — Identification of VapB10 and VapC10-His6 by MS analysis. Both VapB10 (A) and VapC10-His6 (B) from lane 4 in the Figure 3 were confirmed by MS analysis. Shown below are amino acid sequences and predicted m/z values of VapB10 and VapC10 by online analysis using the MS-DIGEST program (http://prospector.ucsf.edu). (TIF) [file pone.0080716.s003.tif]
