# Supplementary material for: Transcriptional and Proteolytic Regulation of the Toxin-Antitoxin Locus vapBC10 (ssr2962/slr1767) on the Chromosome of Synechocystis sp. PCC 6803
Source: PLoS One. 2013 Nov 19;8(11):e80716. doi: 10.1371/journal.pone.0080716 (PMC3834315; doi:10.1371/journal.pone.0080716)
Supplement: Table S1 — The putative vapBC TA loci on the Synechoycystis chromosome. (DOCX) [file pone.0080716.s004.docx]

**Table S1 The putative *vapBC* TA loci on the *Synechoycystis* chromosome**

| TA loci^a^ | Gene pair | Dis.^b^ | Protein GIs | Antitoxin/toxin length (amino acids) ^c^ | Protein domain | Reference |
| --- | --- | --- | --- | --- | --- | --- |
| *vapBC1* | *ssl2922/ssl2923* | 0 | 16329605, 16329604 | 75/ 83 | AbrB/PIN | [1,2] |
| *vapBC2* | *sll1714/sll1715* | -68 | 16330048/16330040 | 133/157 | No CD/PIN | [1] |
| *vapBC3* | *ssl2420/sll1225* | 9 | 16330685/16330680 | 84/149 | No CD/PIN | [1] |
| *vapBC4* | *ssl2138/sll1092* | -1 | 16330930/16330920 | 68/128 | COG5559/PIN | [1,2] |
| *vapBC5* | *-/ssr0756* | - | 16329208 | -/61 | PIN | [1] |
| *vapBC6* | *ssl0385/sll0205* | 3 | 384437485/16331422 | 84/125 | COG5559 /PIN | [1,2] |
| *vapBC7* | *ssl1300/sll0690* | 2 | 1,633,153,316,331,530 | 85/133 | AbrB/PIN | [1] |
| *vapBC8* |  | -79 | c3044422-3044670/ c3044058-3044501 | 83/148 |  | [1] |
| *vapBC9* | *ssl1004/sll0525* | 11 | 16332080/16332070 | 92/131 | PHD/PIN | [1, 2] |
| *vapBC10* | *ssr2962/slr1767* | -3 | 16329657/16329658 | 74/112 | COG2442/PIN | [2] |
| *vapBC11* | *slr1209/slr1210* | 7 | 16329983/16329984 | 109/ 120 | COG2442/PIN | [2] |
| *vapBC12* | *ssl2733/sll1400* | -3 | 16329208/16329207 | 76/113 | COG2442/PIN | [2] |
| *vapBC13* | *slr0770/slr0771* | -17 | 16331314/16331315 | 106/111 | COG2442/PIN | [2] |
| *vapBC14* | *sll0658/ssl1255* | -3 | 16331968/16331967 | 89/164 | COG2886/PIN | [2] |
| *vapBC15* | *ssr2201/slr1327* | -10 | 1653164/16330673 | 72/144 | No CD/PIN | unpublished |
| *vapBC16* | *ssl3615/sll1912* | -3 | 1652356/16329870 | 96/124 | No CD/PIN | unpublished |

Footnotes:

a) The TA loci *vapBC-1*_*vapBC-9* were named by Pandey [1]. The remaining *vapBC* loci were reported by Makarova [2] and are named here. The *vapBC-5* locus only contains a solitary *vapC*. The ORFs of the *vapBC-8* locus were found by Pandey [1] but is not annotated in cyanobase (<http://genome.microbedb.jp/cyanobase>). The *vapBC-15* and *vapBC-16* loci were identified by our laboratory (unpublished)

b) Dis. gives the distance (in bp) between the 3' end of the antitoxin gene and 5' end of the toxin gene. A negative symbol indicates overlap between the genes.

c) The lengths of the antitoxin and toxin proteins are given by the number of codons predicted from the DNA.

**References**

1. Pandey DP, Gerdes K (2005) Toxin-antitoxin loci are highly abundant in free-living but lost from host-associated prokaryotes. Nucleic Acids Res 33: 966-976.

2. Makarova K, Wolf Y, Koonin E (2009) Comprehensive comparative-genomic analysis of Type 2 toxin-antitoxin systems and related mobile stress response systems in prokaryotes. Biol Direct 4: doi:10.1186/1745-6150-1184-1119.
