# Supplementary material for: Transcriptional and Proteolytic Regulation of the Toxin-Antitoxin Locus vapBC10 (ssr2962/slr1767) on the Chromosome of Synechocystis sp. PCC 6803
Source: PLoS One. 2013 Nov 19;8(11):e80716. doi: 10.1371/journal.pone.0080716 (PMC3834315; doi:10.1371/journal.pone.0080716)
Supplement: Table S2 — The PCR primers used in this study. (DOCX) [file pone.0080716.s005.docx]

**Table S1. The PCR primers used in this study**

| primer | sequence (5′→3′) ^a^ | Restriction enzyme | Uassage |
| --- | --- | --- | --- |
| ssr2962-R | GGATTCGAGTGAGCGACATCC |  | RT-PCR |
| slr1767-R1 | CGTAACGTTACCGCAAGTTAGCC |  | RT-PCR and RACE |
| slr1767-R2 | CAGAGTTGGCAACTCGTGCGG |  | RACE |
| slr1767-R3 | CCCGTAATCCCAGATCCCTC |  | RACE |
| ssr2962-S | ggagaGAGCTCGAATAGCTTACTGTCTCGCATTAC | *Sac*I | Selection expression |
| Ssr2962-K | gGAGAGGTACCGTAATCCCAGATCCCTC | *Kpn*I | Selection expression and *lacZ*-fusion |
| Slr1767-N | GGAGAACATATGAATATTTGGGTTGATGCTC | *Nde*I | Selection expression |
| Slr1767-K | ggagaaggTACCTACTGCAATCGGGCTGG | *Kpn*I | Selection expression and co-expression |
| Ssr2962-N | GgagacatatGAATAGCTTACTGTCTCGCATTAC | *Nde*I | Co-expression |
| slr1767-X | GGAGAACTCGAGGGAGTTGCTGATTTCTATGATA | *Xho*I | Co-expression and *lacZ*-fusion |
| P_vapBC10_-1 | GAGGAAGGATCCGGAACAGCCGGTGGCTTCCG | *Bam*HI | *lacZ*-fusion |
| Ssr2962-B1 | ggagaGGATCCGAATAGCTTACTGTCTCGCATTAC | *Bam*HI | *lacZ*-fusion |
| Ssr2962-B2 | GGAGAAGGATCCGTAATCCCAGATCCCTC | *Bam*HI | *lacZ*-fusion |
| Slr1767-B | ggagaaggATCCGGAGTTGCTGATTTCTATGATA | *Bam*HI | *lacZ*-fusion |
| P_vapBC10_-E1 | CGCTTCACTGAGCTGGTTGAGG |  | EMSA |
| P_vapBC10_-E2 | GAGAAGGATCCGATGTCGCTCACTCGAATCC | *Bam*HI | EMSA and *lacZ*-fusion |
| P_vapBC10_-E3 | GGAGATTAACTTTCCCTTCAG |  | EMSA |
| P_vapBC10_-E4 | GGACGACCACCACACTGACC |  | EMSA |
| P_vapBC10_-E5 | CTGACTGACTTTTGATACGG |  | EMSA |
| P_vapBC10_-E6 | CACTCGAATCCTCATCCCTCG |  | EMSA |
| P_vapBC10_-E7 | GGAGAAAATCAAAAAATGCGATGTAG |  | EMSA |
| P_BAD_-F | gtccacattgattatttgcacgg |  | EMSA |
| P_BAD_-R | CTTCTCCTTTGCTAGCCATATG |  | EMSA |
| lacZ-R | ctgcgcaactgttgggaagg |  | *lacZ*-fusion |
| slr0168-1 | GATATCGTTCCATCGCCGCCCAC |  | *lacZ*-fusion |
| slr0168-2 | GATTGGTGGCTAACCAGAGG |  | *lacZ*-fusion |

Footnote:

1. The underlined bases represent recognition sites for specific restriction enzymes listed in the column “Restriction enzyme”.
